# Intra-rater and inter-rater reliability of six musculoskeletal preparticipatory screening tests

**DOI:** 10.4102/sajp.v75i1.469

**Published:** 2019-04-24

**Authors:** Nosipho Zumana, Benita Olivier, Lonwabo Godlwana, Candice Martin

**Affiliations:** 1 Department of Physiotherapy, University of the Witwatersrand, Johannesburg, South Africa

## Abstract

**Background:**

High injury prevalence rates call for effective sports injury prevention strategies, which include the development and application of practical and reliable pre-participatory screening tools.

**Objectives:**

The aim of this study was to investigate the intra-rater and inter-rater reliability of the one-legged hyperextension test (1LHET), the empty can (EC) and full can (FC) tests, the standing stork test (SST), the bridge-hold test (BHT) and the 747 balance test (747BT).

**Method:**

Thirty-five healthy, injury-free male athletes (cricket and soccer players), aged 16–24 years, were evaluated by two physiotherapists. For each of the tests, the participants were evaluated twice (on two consecutive days) by each physiotherapist. Both the intra- and inter-rater reliability were determined. Cohen’s kappa (*k*) was calculated for the 1LHET, the EC and FC tests and the SST. The intraclass correlation coefficient (ICC) was used for the BHT and the 747BT. A confidence level of 95% (*p* ≤ 0.05) was applied as the criterion for determining the statistical significance of the results.

**Results:**

The SST presented with the lowest level of intra-rater agreement (ICC = –0.20 to 0.10). On the other hand, the EC test was the only test where one rater achieved an excellent intersessional agreement (*k* = 0.80; 95% confidence interval [CI] 0.40–1.20). Substantial to excellent results for the inter-rater agreement for both sessions were recorded for the 1LHET (*k* = 0.70–0.90) and the BHT (ICC = 0.70–0.90).

**Conclusion:**

Reliability values need to be considered when making clinical decisions based on screening tests. A more refined description of the testing procedures and criteria for interpretation might be necessary before including the six screening tests investigated in this study in formal screening protocols.

**Clinical implication:**

Confirmed reliability of screening tests would enable sports professionals to make informed decisions when designing preparticipatory musculoskeletal screening tools and when dealing with the management of injury risks in athletes.

**Keywords:**

musculoskeletal screening; injury risk management; intra-rater reliability; inter-rater reliability; soccer; cricket.

## Introduction

In South Africa, soccer and cricket remain popular sports. Injury prevalence studies highlight that musculoskeletal injuries are inevitably a component in the career of the professional soccer (Naidoo 2007) and cricket (Stretch 2001:336) player. Naidoo (2007) reported that over a competitive season, the majority (57%) of soccer players in a professional South African team were found to have sustained injuries. Lower limb injuries were most prevalent among defenders and midfielders, while goalkeepers and forwards were more prone to injuries of the trunk (Naidoo 2007). Cricket injury prevalence rates pose an equal challenge.

Stretch (2001) conducted a 3-year longitudinal study and concluded that cricketers tend to be more prone to lower limb injuries (49.50%), followed by injuries to the upper limbs (23.30%), back and trunk (22.80%). Bowling accounts for more injuries (41.3%) than fielding, including wicketkeeping (28.6%) and batting (17.1%).

These high injury rates call for effective sports injury prevention strategies, which include the development and application of preparticipatory screening tools (Madsen, Drezner & Salerno 2014:142). The ultimate goals of musculoskeletal screening are to identify the modifiable and non-modifiable risks to injury, to facilitate optimal musculoskeletal health and to optimise performance (Cook, Burton & Hoogenboom 2006:62; Ekstrom, Donatelli & Carp 2007:754; Lehr et al. 2013:225).

Tests included in preparticipation screening tools should be practical and reliable. These tests should enable health professionals, including physiotherapists, to determine the athlete’s musculoskeletal condition and risk of injury. A screening test is considered to be reliable if there is an error-free consistency, whereby the test measurements can be reproduced by two different raters (inter-rater reliability) and repeatedly by the same rater (intra-rater reliability) (Portney & Watkins 2000:768). Agreement between ratings ensures that results are comparable and that accurate conclusions can therefore be drawn from the results.

The tests included in this study, namely the one-legged hyperextension test (1LHET), the empty can (EC) and full can (FC) tests, the standing stork test (SST), the bridge-hold test (BHT) and the 747 balance test (747BT), attempt to identify intrinsic, person-related risk factors. These tests have been included in the screening protocols of the regulatory bodies of different professional sporting teams, including those of the South African National Cricket (Gray 2015) and Rugby teams (Gray & Naylor 2009), as well as that of the International Football Federation’s Medical and Research Centre (Dvorak & Junge 2009). To demonstrate the need for an investigation into the reliability, a brief overview of the literature on each of these tests will follow.

### One-legged hyperextension test

Sporting activities that require repetitive lumbar extension and rotation such as cricket pace bowling predispose athletes to lumbar spondylosis (Masci et al. 2006:940; Wiesel 2018). Moderate sensitivity (50% – 75%) and low specificity (12% – 32%) have been reported in the 1LHET and serve as a means to diagnose spondylolysis (Gregg, Dean & Schneiders 2009:121; Masci et al. 2006). Although results from these validity studies present reasons for conducting further investigations, this test is still included in the preparticipatory screening and diagnostic procedures in sports such as cricket (Gray 2015).

It is important to note, however, that only limited research has been conducted in terms of the reliability of the 1LHET.

### Empty can and full can tests

The subacromial space accommodates, among others, the tendon of the supraspinatus muscle, which is responsible for glenohumeral joint compression, abduction and, to a lesser degree, external rotation.

Supraspinatus activity increases with resisted scapular plane motions (Hughes & Na 1996:75). The EC test (Beaudreuil et al. 2009:15) and the FC test (Kelly, Kadrmas & Speer 1996:581) were designed to identify a supraspinatus tendon pathology that might lead to the encroachment of the subacromial space during activation. Humeral internal rotation, a component of the EC test (Cools, Cambier & Witvrouw 2008:628), blocks greater tuberosity movement, preventing the humerus from giving way under the acromion during its elevation, thus leading to further subacromial space encroachment (Hughes & Na 1996:75). For this reason, the FC test might be favoured above the EC test (Hughes & Na 1996:75). Results from several studies propose that the FC and EC tests demonstrate acceptable diagnostic accuracy, that is sensitivity, specificity and likelihood ratios, for full or partial thickness in supraspinatus tendon ruptures (Itoi et al. 1999:65; Kim et al. 2006:223; Lasbleiz et al. 2014:228; Somerville et al. 2014:1911).

Liu et al. (2016:147) reported sensitivity levels of 84.30% and 78.90% and specificity levels of 74.50% and 80.90% for the EC and FC tests, respectively (Liu et al. 2016:147). Michener et al. (2009:1898) investigated the inter-rater reliability of the EC test and reported a kappa value of 0.45 to 0.67. However, unlike in our study, the inter-rater reliability test was based only on evidence of weakness and disregarded pain as a component (Kelly et al. 1996). No literature specifically reporting on the inter- and intra-rater reliability of the FC and EC tests among physiotherapists, who are often responsible for the preseason screening of players in a team setting, could be found.

### Standing stork test

The optimal function of the lumbo–pelvic–hip complex allows for the effective generation and transfer of forces during athletic activity (Kibler, Press & Sciascia 2006:189). The SST assesses the ability of the pelvis to remain stable as load is transferred between the spine and the limbs (Hungerford et al. 2007:879). Hungerford et al. (2007) investigated the ability of physiotherapists to evaluate intrapelvic movement using the SST and found good inter-rater reliability (*k* = 0.67). Conversely, Tong et al. (2006:464) found poor inter-rater reliability for the SST. However, the sample size was small (*n* = 24) and consisted only of females with lower back pain, which limits the generalisation of findings to other populations.

### Bridge-hold test

The BHT assesses gluteal strength and endurance, as well as the static stability of the trunk and pelvis (Dennis et al. 2008:25). The stability of the core allows for improved balance and for the motion of the trunk over the pelvis (Andrade et al. 2012:268). Andrade et al. (2012) investigated the intra- and inter-rater reliability of the BHT using a two-dimensional motion analysis and reported kappa values of 0.32–0.58 and 0.80, respectively. In the light of the costs and logistics related to two-dimensional motion analysis, there is a need to determine the reliability of the BHT without the application of movement analysis software, which is also often the case in clinical practice.

### 747 Balance test

The 747BT (also known as the ‘Romanian deadlift’) assesses general balance, coordination and stability in a single-leg body position (Strauts & Tate 2015:43) and is, therefore, considered to be applicable to sporting activities that require a combination of strength, flexibility and speed (Gamble 2013). It is important to note, however, that limited research related to the validity and reliability of the 747BT is currently available.

From the literature, it is clear that research related to the reliability of these six screening tests is limited. The intra- and inter-rater reliability of the aforementioned six tests were therefore investigated in order to provide guidance as to the inclusion of these tests in the official musculoskeletal screening protocols of professional sporting teams.

## Materials and methods

This reliability study was conducted at the sports fields of the cricket and soccer clubs of a tertiary institution.

Thirty-five healthy, injury-free male players aged between 16 and 24 years from the university’s respective soccer and cricket clubs were randomly selected for the study. Players with a history of spinal or lower limb surgery were excluded. The sample size was based on the findings and suggestions by Sim and Wright (2005:257). Effect sizes (ES) were calculated using Cohen’s *d*-test, where ES values of 0.20, 0.50 and 0.80 were respectively interpreted as small, medium and large. An a priori power analysis, using G-power relating to the medium ES category (ES = 0.5) was used in the calculation to determine sample size. A power analysis for estimating the size of the sample that would yield a power of 80% was conducted prior to the data collection phase.

### Procedures

Three participants (±10% of the main sample size), other than those included in the main study, were included in the pilot study, which used the same inclusion and exclusion criteria specified for the main study.

The pilot study aimed to familiarise the raters with the testing procedures, to ensure that the testing instructions and procedures were standardised and to establish the time required for the completion of each test. The data collected from the pilot study were not included for the analysis of the main study results as changes to the standardised testing instructions and conditions (i.e. time of day: before, during or after training) had been made to the study procedure subsequent to the pilot study.

The main study was conducted over 2 weeks. To minimise the effect of physiological and biomechanical changes and to allow the symptoms that might have been provoked by the tests to subside, the first and second testing sessions for the individual participants occurred on two consecutive days. The second session for a specific participant occurred under the same conditions (i.e. before, during or after training) as those for the first. The screening tests were conducted by two qualified physiotherapists (Rater 1 and Rater 2), each with more than 5 years of clinical experience. Video recordings were made of each test for digital storage purposes and were in turn managed by a research assistant.

**FIGURE 1:**
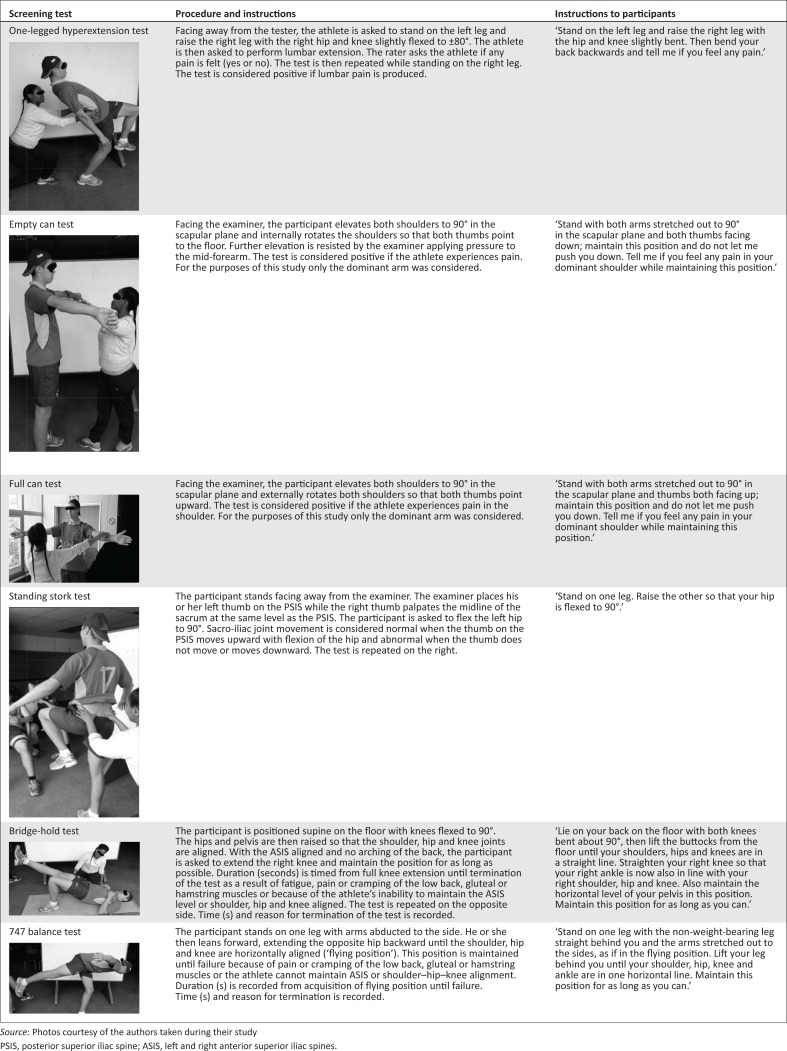
Procedures and standard instructions for the one-legged hyperextension test, full can test, empty can test, standing stork test, bridge-hold test and 747 balance test.

The screening tests were conducted according to a standard set of instructions and procedures (Figure 1) and performed in the following order: 1LHET, BHT, 747BT, ECTFCT, SST, without any period of rest between tests. Each rater assessed each participant. The FC and EC tests and the 1LHET and SST required a ‘hands-on’ assessment by the respective raters and were conducted and rated separately by each of them. Being observational tests, the BHT and the 747BT were rated simultaneously by the raters. During the simultaneous ratings, no communication was allowed between the raters, who were blinded to each other’s findings.

### Data analysis

Data were recorded on specifically designed data collection sheets and later captured by the first author on an Excel spreadsheet. Statistical analyses were accomplished using SPSS Version 23 (IBM Corporation, Armonk, NY, USA). Descriptive analysis was used to describe the basic features of the data.

Agreement in the test results by two different raters (inter-rater reliability) and repeatedly by the same rater (intra-rater reliability) was determined. The inter-rater reliability was determined by comparing the per-session ratings of Rater 1 as opposed to those of Rater 2. Between-day intra-rater reliability was tested by comparing the ratings of a rater for Session 1 with those of the same rater for Session 2. To determine both the inter- and intra-rater reliability, Cohen’s kappa (*k*) was used for the 1LHET, EC and FC tests, and the SST because the outcomes (yes or no) for these tests were nominal (Cohen 1960; Sim & Wright 2005). The intraclass correlation coefficient (ICC_3,2_) was used for the BHT and the 747BT, the data for which were continuous. The ICC was measured through a two-way random effect for inter-rater reliability, and a mixed random effect for intra-rater reliability was used because each participant from this random sample was assessed more than once (Shrout & Fleiss 1979:420). A confidence interval of 95% (*p* < 0.05) was used to determine the statistical significance of the data. The *k* and ICC values were interpreted according to the guidelines as set out by Landis and Koch (1977:159) (Table 1).

**TABLE 1:** Guidelines for interpretation of kappa and intraclass correlation coefficient values.

Value of *k*† or ICC	Strength of agreement
< 0.00	No agreement
0.00–0.20	Slight
0.21–0.40	Fair
0.41–0.60	Moderate
0.61–0.80	Substantial
0.81–1.00	Excellent

*Source*: Landis and Koch 1977:159

† , *k* = kappa; ICC, intraclass correlation coefficient.

### Ethical considerations

Ethical clearance (reference number: M150626) was obtained from the University of the Witwatersrand’s Human Research Ethics Committee (Medical). Each participant received an information leaflet presenting the goals and procedures of the study and was requested to voluntarily provide consent to participate in the study and to permit a video recording of their performance in the respective tests.

## Results

Of the 35 selected participants, four (11%) could not return for the second assessment because of unexpected time conflicts with training and study-related responsibilities. Therefore, data from 31 participants (89%) were eligible for analysis. Table 2 summarises the demographic (age) and anthropometric data of the 31 participants included in the main study.

**TABLE 2:** Demographic and anthropometric data of participants (*n* = 31).

Variable	Mean ± SD	Minimum	Maximum
Age (yr)	18.60 ± 1.50	16.00	24.00
Weight (kg)	74.90 ± 9.40	60.00	100.00
Height (m)	1.80 ± 0.10	1.50	1.90

SD, standard deviation.

The intra-rater reliability results are summarised in Table 3. Only Rater 2’s assessment of the EC test showed substantial intra-rater reliability (*k* = 0.80; 95% confidence interval [CI] 0.40–1.20), while the intra-rater reliability levels for the SST for both raters were poor or slight.

**TABLE 3a:** Intra-rater reliability of the screening tests in this study.

Rater	Test	Session 1 outcomes (*n* = 31)		Session 2 outcomes (*n* = 31)		*k* (95% CI)	SEM
		Yes *n* (%)	No *n* (%)	Yes *n* (%)	No *n* (%)		
	**1LHET**						
1	Left	10.00 (32.30)	21.00 (67.00)	7.00 (22.60)	24.00 (77.40)	0.60 (0.30–0.90)	0.20
	Right	11.00 (35.50)	20.00 (64.50)	5.00 (16.10)	26.00 (83.90)	0.50 (0.20–0.80)	0.20
2	Left	13.00 (11.90)	18.00 (58.10)	6.00 (19.40)	25.00 (80.60)	0.40 (0.10–0.70)	0.20
	Right	11.00 (35.50)	20.00 (64.50)	4.00 (12.90)	27.00 (87.10)	0.40 (0.10–0.70)	0.20
	**EC**						
1	Dominant arm	2.00 (6.50)	29.00 (93.50)	1.00 (3.20)	30.00 (96.80)	0.60 (0.20–1.1)	0.20
2	Dominant arm	0.00 (0.00)	31.00 (100.00)	1.00 (3.20)	30.00 (96.80)	0.80 (0.40–1.20)	0.20
	**FC**						
1	Dominant arm	1.00 (3.20)	30.00 (96.80)	1.00 (3.20)	30.00 (96.80)	0.10 (0.10–0.20)	0.10
2	Dominant arm	1.00 (3.20)	30.00 (96.80)	1.00(3.20)	30.00 (96.80)	0.60 (0.20–1.10)	0.20

1LHET, one-legged hyperextension test; EC, empty can test; FC, full can test; *k*, kappa; SEM, standard error measurement; CI, confidence interval.

**TABLE 3b:** Intra-rater reliability of the screening tests in this study.

Rater	Test	Session 1 outcomes (*n* = 31)			Session 2 outcomes (*n* = 31)			*k* (95% CI)	SEM
		Motion detected by rater			Motion detected by rater				
		Up	Down	None	Up	Down	None
	**SST**								
1	Left	16.00 (51.60)	4.00 (12.90)	11.00 (35.50)	14.00 (45.20)	13.00 (41.90)	3.00 (9.70)	−0.06 (−0.27–0.10)	0.10
	Right	17.00 (54.80)	6.00 (19.40)	8.00 (25.80)	11.00 (35.50)	18.00 (58.10)	2.00 (6.50)	−0.15 (−0.40–0.40)	0.10
2	Left	0.00 (0.00)	16.00 (51.60)	15.00 (48.40)	0.00 (0.00)	22.00 (70.90)	9.00 (29.00)	0.10 (−0.20-0.40)	0.20
	Right	0.00 (0.00)	24.00 (77.40)	7.00 (22.60)	0.00 (0.00)	27.00 (87.10)	4.00 (12.90)	−0.20 (−0.30–0.10)	0.10

*k*, kappa; SEM, standard error measurement; CI, confidence interval.

**TABLE 3c:** Intra-rater reliability of the screening tests in this study.

Rater	Test	Session 1 outcomes (*n* = 31)	Session 2 outcomes (*n* = 31)	ICC_3,2_ (95%CI)	SEM
		Seconds held (mean ± SD)	Seconds held (mean ± SD)		
	**BHT**				
1	Left	26.30 ± 10.80	17.30 ± 12.50	0.30 (−0.40–0.60)	15.70
	Right	21.00 ± 8.70	18.10 ± 10.40	0.50 (0.20–0.70)	11.50
2	Left	22.90 ± 12.40	16.50 ± 12.50	0.40 (0.10– 0.70)	15.80
	Right	18.20 ± 8.50	16.30 ± 10.10	0.40 (0.10–0.70)	11.90
	**747 BT**		
1	Left	17.80 ± 9.10	15.80 ± 10.9	0.60 (0.30–0.80)	11.70
	Right	16.50 ± 9.70	15.20 ± 10.80	0.30 (−0.10–0.60)	13.90
2	Left	15.70 ± 10.10	15.20 ± 8.40	0.40 (0.1–0.7)	11.90
	Right	15.30 ± 7.50	16.40 ± 9.30	0.20 (−0.4–0.7)	11.60

BHT, bridge-hold test; 747BT, 747 balance test; ICC, intraclass correlation coefficient; SEM, standard error measurement; CI, confidence interval.

The inter-rater reliability levels for each of the screening tests included are shown in Table 4. Notably, with the exception of the SST, the left BHT and the left 747BT, the inter-rater agreement always tended to be higher during Session 2, and the agreement between the results for this session for the EC test (*k* = 0.80; 95% CI 0.40–1.20), the FC test (*k* = 0.80; 95% CI 0.50–1.10) and the right BHT (ICC = 0.80; 95% CI 0.60–0.90) was substantial. Only the 1LHET (bilaterally) revealed substantial to excellent agreement for both sessions. A poor agreement between the raters was noted for the EC test for Session 1 (*k* = –0.05; 95% CI –0.10 to 0.01) and for the SST (right) for Session 2 (*k* = -0.06; 95% CI –0.20 to 0.10).

**TABLE 4a:** Inter-rater reliability of the screening tests in this study.

Session	Test	Rater 1 outcomes (*n* = 31)		Rater 2 outcomes (*n* = 31)		*k* (95% CI)	SEM
		Yes *n* (%)	No *n* (%)	Yes *n* (%)	No *n* (%)		
	**1LHET**						
1	Left	10.00 (32.30)	21.00 (67.70)	13.00 (41.90)	18.00 (58.10)	0.80 (0.60–1.00)	0.1
	Right	11.00 (35.50)	20.00 (64.50)	11.00 (35.50)	20.00 (64.50)	0.80 (0.60–1.00)	0.1
2	Left	7.00 (22.60)	24.00 (77.40)	6.00 (19.40)	25.00 (80.60)	0.90 (0.70–1.10)	0.1
	Right	5.00 (16.10)	26.00 (83.90)	27.00 (87.10)	0.90 (0.60–1.10)	0.90 (0.60–1.10)	0.1
	**EC**						
1	Dominant arm	2.00 (6.50)	29.00 (93.50)	0.00 (0.00)	31.00 (100.00)	−0.05 (−0.10–0.01)	0.003
2	Dominant arm	1.00 (3.20)	30.00 (96.80)	1.00 (3.20)	30.00 (96.80)	0.80 (0.40–1.20)	0.20
	**FC**						
1	Dominant arm	1.00 (3.20)	30.00 (96.80)	1.00 (3.20)	30.00 (96.80)	0.50 (0.03–0.90)	0.20
2	Dominant arm	1.00 (3.20)	30.00 (96.80)	1.00 (3.20)	30.00 (96.80)	0.80 (0.50–1.10)	0.10

1LHET, one-legged hyperextension test; EC, empty can test; FC, full can test; *k*, kappa; SEM, standard error measurement; CI, confidence interval.

**TABLE 4b:** Inter-rater reliability of the screening tests in this study.

Session	Test	Rater 1 outcomes (*n* = 31)			Rater 2 outcomes (*n* = 31)			ICC_3,2_ (95% CI)	SEM
		Motion detected by rater			Motion detected by rater				
		Up	Down	None	Up	Down	None		
	**SST**								
1	Left	16.00 (51.60)	4.00 (12.90)	11.00 (35.50)	0.00 (0.00)	16.00 (51.60)	15.00 (48.40)	0.30 (0.10–0.40)	0.10
	Right	17.00 (54.80)	6.00 (19.40)	8.00 (25.80)	0.00 (0.00)	24.00 (77.40)	7.00 (22.60)	0.10 (−1.10–1.30)	0.60
2	Left	14.00 (45.20)	13.00 (41.90)	3.00 (9.70)	0.00 (0.00)	22.00 (70.90)	9.00 (29.00)	0.30 (0.20–0.40)	0.10
	Right	11.00 (35.50)	18.00 (58.10)	2.00 (6.50)	0.00 (0.00)	27.00 (87.10)	4.00 (12.90)	−0.06 (−0.20–0.10)	0.10

SST, standing stork test; ICC, intraclass correlation coefficient; SEM, standard error measurement; CI, confidence interval.

**TABLE 4c:** Inter-rater reliability of the screening tests in this study.

Session	Test	Rater 1 outcomes (*n* = 31)	Rater 2 outcomes (*n* = 31)	ICC3,2 (95% CI)	SEM
		Seconds held (mean ± SD)	Seconds held (mean ± SD)		
	**BHT**				
1	Left	26.30 (±10.80)	22.90 (±12.40)	0.80 (0.70–0.90)	9.10
	Right	21.00 (±8.70)	18.20 (±8.50)	0.70 (0.40–0.80)	9.20
2	Left	17.30 (±12.50)	16.50 (±12.50)	0.90 (0.80–0.90)	6.90
	Right	18.10 (±10.40)	16.30 (±10.10)	0.80 (0.60–0.90)	9.40
	**747 BT**		
1	Left	17.80 ± 9.10	15.70 ± 10.10	0.70 (0.50–0.90)	9.30
	Right	16.50 ± 9.70	15.30 ± 7.50	0.20 (0.00–0.70)	11.30
2	Left	15.80 ± 10.90	15.20 ± 8.40	0.40 (0.10–0.70)	11.90
	Right	15.20 ± 10.80	16.40 ± 9.30	0.20 (−0.20–0.50)	11.60

BHT, bridge-hold test; 747BT, 747 balance test; ICC, intraclass correlation coefficient; SEM, standard error measurement; CI, confidence interval.

## Discussion

Sporting teams often include preparticipatory screening tools as part of their injury prevention strategies (van Mechelen, Hlobil & Kemper 1992:82). Reliable, cost- and time-effective screening tools might allow medical and fitness professionals to make informed decisions regarding the management of an athlete’s injury risk. The purpose of this study was therefore to investigate the reliability of six screening tests often included in the screening protocols of various sporting disciplines.

Among other factors, body composition and specific physical attributes have been related to elite and sub-elite level cricketers (Koley 2011:427; Stuelcken, Pyne & Sinclair 2007:1587) and soccer players (Hencken & White 2006:205). Considering the mean age and level of participation, the weight and height measurements of the participants were similar to those of the cricketers (21.03 ± 1.72 years; 61.83 ± 9.6 kg; 171.00 ± 7.1 cm) (Koley 2011) and soccer players (66.60–78.00 kg; 171.2–178.1 cm) (Rebelo et al. 2012:312) investigated in other studies. Although body composition and specific physical characteristics have been associated with advanced performance in general athletic and sport-specific skills (Rodriguez, DiMarco & Langley 2009), these specifics do not fall within the scope of this study. A summation of the intra- and inter-rater reliability results of the screening tests investigated in this study are presented in Table 5.

**TABLE 5:** Summation of strength of intra- and inter-rater agreement for the screening tests included in this study.

Test	Side	Intra-rater reliability		Inter-rater reliability	
		Rater 1	Rater 2	Session 1	Session 2
1LHET	Left	Moderate	Fair	Substantial	Excellent
	Right	Moderate	Fair	Substantial	Excellent
EC	Dominant	Moderate	Substantial	No agreement	Substantial
FC	Dominant	Slight	Moderate	Moderate	Substantial
SST	Left	No agreement	Slight	Fair	Fair
	Right	No agreement	No agreement	Slight	No agreement
BHT	Left	Fair	Fair	Substantial	Excellent
	Right	Moderate	Fair	Substantial	Substantial
747BT	Left	Moderate	Fair	Substantial	Fair
	Right	Fair	Slight	Slight	Slight

1LHET, one-legged hyperextension test; EC, empty can; FC, full can; SST, standing stork test; BHT, bridge-hold test; 747BT, 747 balance test.

### One-legged hyperextension test

While the 1LHET was the only test presenting with substantial to excellent inter-rater agreement in this study, the intra-rater agreement was moderate (Rater 1) to fair (Rater 2). This was also the only bilateral test (i.e. performed on the left and right sides) in which both raters achieved the same level of intersessional agreement for the left and right sides. This might indicate that the test was performed in a uniformly bilateral manner by each rater during Session 1 and Session 2 but that the level of pain experienced by the participants during the respective sessions differed.

Another explanation could be related to the lack of specification in terms of the lumbar extension range according to which the test was performed. The designers of the 1LHET hypothesised that in the presence of spondylolysis, compressive forces on the pars interarticularis, associated with lumbar extension, would exacerbate the pain (Jackson et al. 1981:304). A specific lumbar spine extension range was not described, however, and was therefore apparently left to the discernment of the examiner. During the execution of the test, a manipulation of the lumbar extension range by the participant from one assessment session to the next, as well as the resultant change in compression of the pars interarticularis, might account for different levels, if any, of pain.

Despite the substantial to excellent inter-rater reliability measured in this study, the less-than-substantial intra-rater reliability and conclusions from studies investigating the validity of the 1LHET (Alqarni et al. 2015:268; Masci et al. 2006:940) place doubt on its usefulness as the first-line pathognomonic test for spondylosis.

### Empty can test

In this study, the intra-rater reliability of the EC test proved to be moderate to substantial, with a small standard error measurement (SEM) (0.20), which indicates a higher level of rater agreement compared to that for the 1LHET, specifically in respect of Rater 2. Limited research related to the intra-rater reliability of the EC test has been conducted. As such, a comparison of the results in this study proved to be difficult. However, other studies investigating the diagnostic accuracy of the EC test have reported moderate (*k* = 0.4–0.43 [0.13–0.67]) inter-rater reliability (Magee, Sueki & Chepeha 2011; Michener et al. 2009:1898). Our study, however, found no agreement between the ratings of Raters 1 and 2 for Session 1 but substantial agreement between their respective ratings for Session 2. However, the range for the 95% confidence level for both sessions was broad and the inter-rater kappa values should therefore be interpreted with caution. The limited homogeneity of the rater outcomes for a screening test might highlight the defects of the screening tools or suggest that the raters require additional training in the use of the tool (Martin &Altman 1986:307).

In another study investigating the inter-rater reliability of, among others, the EC test, the outcomes of a research nurse (with no formal musculoskeletal training) and a specialist consultant (a rheumatologist with a special interest in shoulders), as well as the outcomes of the same research nurse and specialist rheumatology registrar, reported fair inter-rater agreement (*k* = 0.38–0.46) (Ostor 2004:1288). These results might indicate that regardless of the expertise of the examiner (expert vs. expert or novice vs. expert), the inter-rater agreement for the EC test was at most moderate. In our study, however, regardless of similar examiner qualifications and experience, the difference in the level of rater agreement between the two sessions was noteworthy (no agreement for Session 1 vs. substantial agreement for Session 2). One might therefore infer that additional training in the execution of the EC test and in the interpretation of the test results might be warranted.

### Full can test

Prior to this study, research investigating the reliability of the FC test had not been documented (Gray 2015), making the comparison of results challenging. However, the validity of the FC test in the diagnosis of supraspinatus pathology has been confirmed by several studies (Itoi et al. 1999:65; Kelly et al. 1996:581). In our study, intra-rater reliability was found to be slight and moderate for Raters 1 and 2, respectively. On the other hand, inter-rater agreement proved to be moderate to substantial. One explanation for the differences in agreement between the respective sessions, as well as between the raters, might be related to differences in the symptoms experienced by the participants. Another might be on account of a variation in the amount of resistance applied by the raters, which in turn elicits varying levels of isometric muscle activity and possible symptoms.

### Standing stork test

No intra-rater agreement was found for Session 1, while Rater 2 found only slight agreement for right-sided sacro-iliac joint (SIJ) dysfunction in Session 2. Inter-rater agreement was at most fair. Reasons for this less-than-optimal reliability level may include the observational and palpatory nature of this test. Compared to pain provocation test results, palpatory SIJ test results show moderate inter-rater agreement (*k* = –0.60) (Robinson et al. 2007:72). This is not unique to SIJ-related testing as similar difficulties have been reported for Craig’s test, which requires the palpation of the greater trochanter for the measurement of femoral anteversion (Choi & Kang 2015:1141).

Like in our study, Hungerford et al. (2007:879) investigated the ability of three physiotherapists to assess SIJ movement using the SST. The authors found that when bone motion (movement of the innominate bone on the sacrum) was recorded on the basis of a two-point scale (occurrence or non-occurrence of bone motion), the agreement between the therapists on intrapelvic motion, which occurs during load transfer, proved to be substantial (*k* = 0.67–0.77) (Hungerford et al. 2007).

However, the use of a three-point scale that is innominate – remains neutral, moves up or moves down – brought moderate reliability (*k* = 0.59) for both the left and the right sides to light (Hungerford et al. 2007:879). The difference in rater agreement using a three-point scale, as was the case for both this and the last-mentioned study, might be a result of the number of physiotherapists assessed. This means that the use of more examiners might result in higher inter-rater reliability levels. Research confirming the association between the level of inter-rater reliability and the number of examiners assessed is yet to be conducted. Tong et al. (2006:464) reported fair inter-rater agreement (*k* = 0.27) between two physiotherapists with regards to the bone motion of the SIJ during testing.

Considering our results and those of the studies mentioned, the reliability of the SST seems dependent on the outcome measure (a two- or a three-point scale) used. Currently, the lack of uniformity in the SST outcome measures and the low measure of reliability of the SST do not justify the inclusion of this test in formal screening procedures.

### Bridge-hold test

The intra-rater reliability for the BHT was found to be fair to moderate, which is similar to the results obtained by Dennis, Elliott and Farhart (2008:25) and Andrade et al. (2012:268), who reported an intra-rater reliability of ICC = 0.56 (95% CI: 0.00, 0.83) and Kw = 0.32–0.58, respectively. The SEM (11.50–15.80) related to the intra-rater reliability in our study points to a large number of errors that might have occurred during testing. This is not surprising considering the observational nature of the test and the number of reasons for terminating it.

Andrade et al. (2012:268) attempted to minimise the subjective component of observational tests to some extent by using two-dimensional motion analyses requiring participants to maintain the unilateral bridge position for a fixed time (10 s) and limiting the test outcomes to the participants. The intra-rater agreement on the ability of the participants to maintain the horizontal alignment of the anterior superior iliac spine for termination still brought only moderate agreement. Numerous studies investigating the reliability of observational musculoskeletal tests that require the assessment of more than one component have been found to have low intra-rater reliability levels (Monnier et al. 2012:1471; Moreland et al. 1997:200; Whatman et al. 2015:210). The BHT also assesses numerous physical fitness aspects such as motor control, endurance, strength and so on, which could be influenced by several factors including training type and intensity and nutritional intake, in a 24-h window period.

The inter-rater reliability of the BHT in this study was substantial to excellent. Andrade et al. (2012:268) reported substantial reliability (Kw = 0.80), while Dennis et al. (2008:25) (ICC = 0.56) reported only moderate inter-rater reliability. The examiners in the latter study assessed the video-recorded performances of the participants in the BHT in separate cubicles as opposed to collectively and simultaneously in one particular facility. This could possibly be the reason for the difference in the inter-rater reliability between the Dennis et al. (2008:25) study and our findings. Our results might indicate that although there is a strong case for inter-rater reliability, the technicalities behind the BHT might require more refined criteria to be applied in the termination phase of the test.

### 747 Balance test

Moderate or less-than-moderate intra-rater reliability was recorded. The inter-rater reliability of the 747BT varied from slight to substantial. Substantial agreement was related only to Session 1’s screening of the left side. To the authors’ knowledge, this was the first study to investigate the reliability of the 747BT. Therefore, it was not possible to compare these results with those of other studies.

Noteworthy, however, are the large SEM values associated with the inter- and intra-rater reliability ICC values.

Like the BHT, the 747BT has numerous test termination criteria and challenges numerous physical fitness components, which could explain the lower level of intra-rater reliability and the large SEM values. Moreover, this is an observational test that was done in real time – similar to what happens in clinical practice – without using video footage or two-dimensional motion analysis, which perhaps allow for greater human error and lower agreement in the sessional observations.

Studies assessing the reliability of real-time observational data have reported poor intra- and/or inter-rater reliability in respect of the various musculoskeletal screening tests (DiMattia et al. 2005:108; Nilstad et al. 2014:358; Örtqvist et al. 2011:2060). Because the two raters evaluated the 747BT simultaneously, it should be kept in mind that their visual vantage points were different, as they could not stand in the exact same spot, which could influence their observations of movement.

We used the recommendations for interpretation of reliability results by Landis and Koch (1977:159) (Table 1). These cut-off values are arbitrary, as no absolute descriptions are possible; however a test with a moderate rating (0.41–0.60) is generally not considered accurate, and results from all screening tests should always be interpreted together with other findings that form part of the holistic assessment of the athlete.

More research is needed in terms of the reliability of clinical tests before they are included in formal screening protocols. Considering our findings, as well as those of other referenced authors, clear instructions in terms of testing procedures and positive test criteria might improve the reliability of the tests. Whatman et al. (2015:210) noted the importance of accurate observational skills in the clinicians responsible for the musculoskeletal evaluations because they allow for instantaneous results in terms of an athlete’s physical condition and performance.

Future research should therefore focus on investigating the effect of more refined testing procedures on the reliability of the screening tests. The fact that our study involved only physiotherapists might make for its limited practical value because the athletes were not also assessed by other medical and fitness professionals.

## Conclusion

The intra-rater reliability of the EC test proved to be moderate to substantial, while the respective values for all of the other tests showed moderate intra-rater reliability to no agreement. The inter-rater reliability of the 1LHET and the BHT, respectively, proved to be substantial to excellent, whereas the other tests performed less satisfactorily in terms of this criterion. Results from the BHT and the 747BT suggest that in order to be reproduced optimally, observational tests should be based on simplified but clearly defined test termination criteria.
